# The Influence of Diabetes Mellitus on the Risks of End-Stage Kidney Disease and Mortality After Liver Transplantation

**DOI:** 10.3389/ti.2022.10023

**Published:** 2022-02-07

**Authors:** Chung-Ying Lee, Mei-Yi Wu, Hsiu-Chen Chan, Tzu-Ting Chen, Le-Yin Hsu, Mai-Szu Wu, Yih-Giun Cherng

**Affiliations:** ^1^ Division of Gastroenterology, Department of Internal Medicine, Shuang Ho Hospital, Taipei Medical University, New Taipei City, Taiwan; ^2^ Division of Gastroenterology, Department of Internal Medicine, School of Medicine, College of Medicine, Taipei Medical University, Taipei, Taiwan; ^3^ Division of Nephrology, Department of Internal Medicine, Shuang Ho Hospital, Taipei Medical University, New Taipei City, Taiwan; ^4^ Division of Nephrology, Department of Internal Medicine, School of Medicine, College of Medicine, Taipei Medical University, Taipei, Taiwan; ^5^ Institute of Epidemiology and Preventive Medicine, College of Public Health, National Taiwan University, Taipei, Taiwan; ^6^ TMU Research Center of Urology and Kidney, Taipei Medical University, Taipei, Taiwan; ^7^ Department of Pharmacy, Shuang Ho Hospital, New Taipei City, Taiwan; ^8^ Center for Neuropsychiatric Research, National Health Research Institutes, Miaoli, Taiwan; ^9^ Graduate Institute of Clinical Medicine, College of Medicine, Taipei Medical University, Taipei, Taiwan; ^10^ Department of Anesthesiology, Shuang Ho Hospital, Taipei Medical University, New Taipei City, Taiwan; ^11^ Department of Anesthesiology, School of Medicine, College of Medicine, Taipei Medical University, Taipei, Taiwan

**Keywords:** liver transplantation, diabetes mellitus, risk, end-stage kidney disease, mortality

## Abstract

This retrospective study aimed to investigate the effect of diabetes mellitus (DM) on the risks of end-stage kidney disease (ESKD) and post-liver transplantation (post-LT) mortality. Using data from the National Health Insurance Research Database, Taiwan, 3,489 patients who received a LT between 1 January 2005, and 31 December 2015, were enrolled in this study and divided into the pre-existing DM, post-LT DM (PLTDM), and without DM groups. All subjects were followed up from 1 year after LT to the index date for ESKD, and the occurrence of death, or until 31 December 2016. Of the 3,489 patients with LT, 1,016 had pre-existing DM, 215 had PLTDM, and 2,258 had no DM pre- or post-LT. The adjusted HRs of ESKD were 1.77 (95% Confidence Interval [CI], .78–3.99) and 2.61 (95% CI, 1.63–4.18) for PLTDM group and pre-existing DM group compared to without DM group, respectively. For the risk of death, the adjusted HRs were 1.05 (95% CI, .72–1.55) and 1.28 (95% CI, 1.04–1.59) for PLTDM group and pre-existing DM group compared to those without DM group, respectively. The sensitivity analysis for the risk of ESKD and death also revealed the consistent result. Pre-existing DM has significant increase the risk of post-LT ESKD and mortality. The role of PLTDM should be explored to explain postoperative morbidity and mortality.

## Graphical Abstract

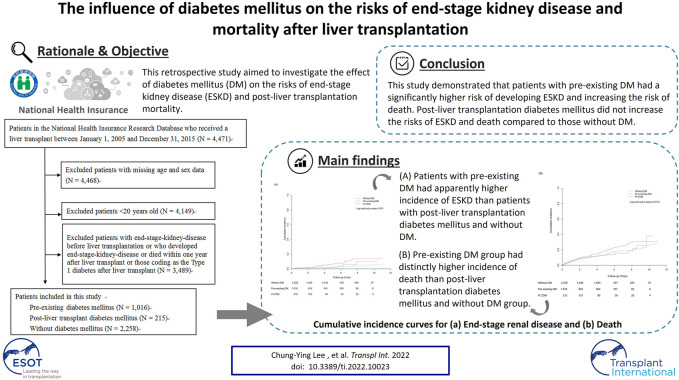

## Introduction

Liver transplantation (LT) is an effective strategy for treating patients with end-stage liver disease and some types of hepatocellular carcinomas (1). With the advancements in surgical techniques and the use of immunosuppressants, patient survival rates have improved globally, reaching nearly 85% at 1 year and 73% at 5 years in Europe and 88% at 1 year and 70% at 5 years in the United States (2). A recent population-based study in Taiwan revealed that the overall 1-year and 5-year survival rate post-LT was 85.1% and 79.6%, respectively (3). An improvement in the early post-LT survival rate underscores the importance of understanding the causes and risk factors for late post-LT mortality.

Renal dysfunction is common in recipients of liver transplant and is a known risk factor for mortality in patients who have undergone LT (4, 5). Cohen et al. reported that 27.5% of LT patients had severe renal dysfunction (measured glomerular filtration rate <40 ml/min/1.73 m^2^) at 5 years with a cumulative incidence of end-stage kidney disease (ESKD) of 6.25% at 7 years and 10% at 10 years (6). Moreover, studies have shown that pre-existing diabetes mellitus (DM), associated with microvascular and macrovascular complications, may influence post-LT morbidity and mortality (7, 8). Post-LT DM (PLTDM) develops in up to 30% of liver transplant recipients, negatively affecting long-term survival (9). However, conflicting results on the effect of PLTDM on post-LT mortality rates exist (10). The relatively limited number of studies examining the impact of pre-existing DM and PLTDM on long-term renal outcomes and mortality, especially the risks of ESKD, prompted us to conduct this retrospective study to investigate the influence of DM on the risks of ESKD and all-cause mortality post-LT by using patient data from the National Health Insurance Research Database (NHIRD) in Taiwan.

## Methods

### Data Collection

We conducted a Nationwide population-based retrospective cohort study using data from the NHIRD, Taiwan. Taiwan initiated its National Health Insurance (NHI) program in 1995. The system covered almost 99% of the entire population in 2007. Taiwan’s population in 2015 was approximately 23 million and the more than 99% of the population is covered by the NHI program. De-identified and computerized data were provided by the National Health Insurance Administration, which organizes claims data for NHI and established the NHIRD. The NHIRD contains basic patient information and medical data from medical claims, including clinical diagnostic codes based on the International Classification of Disease, Revision 9, Clinical Modification (ICD-9-CM). According to the guidelines of the NHI program, the diagnosis code for LT would have been entered by a qualified gastroenterologist or transplant surgeon. The study adhered to the ethical standards of the 2000 Declaration of Helsinki and the Declaration of Istanbul 2008. No executed prisoners were used as donors.

### Study Population and Study Design

The recipients were identified from the NHIRD database using the LT surgery code (codes 75020A or 75020B) from 1 January 2005, to 31 December 2015. We excluded patients with missing age and sex data, who were <20 years old at the time of surgery, who had been diagnosed with ESKD before LT, or who had been coding as the Type 1 diabetes after LT. We also excluded patients who had developed ESKD or died within 1 year after LT to reduce the immortal time bias. The recipients were divided into three groups: pre-existing DM, PLTDM, and without DM. DM (ICD-9-CM code: 250) was identified from medical notes recorded either three or more times in the outpatient department or one or more times in the inpatient department within 1 year before the index date of LT. PLTDM group was defined as those diagnosed as having DM after LT within 1 year. After these three groups were defined, all subjects were start followed from 1 year after LT to the index date for ESKD, the occurrence of death, or until 31 December 2016 to evaluate the risk of ESKD. To estimate the risk of death, all subjects were followed from 1 year after LT to the occurrence of death or until 31 December 2016. We showed our detailed main study design for ESKD and death in Supplementary Figures S1, S2, respectively.

### Outcomes

The primary outcomes in this study are ESKD and death. Patients who had been diagnosed with ESKD were identified when the use of hemodialysis codes (58001C, 58014C, 58019C, 58020C, 58021C, 58022C, 58023C, 58024C, 58025C, 58027C, 58029C, 58030B, 69006C) was more than 24 times in three consecutive months and peritoneal dialysis codes (58002C, 58009B, 58010A, 58010B, 58011A, 58011AB, 58011B, 58011C, 58012A, 58012B, 58017B, 58017C, 58028C) was more than three consecutive months or renal transplantation surgery (76020A, 76020B) was performed. Mortality data were obtained from the Taiwanese Ministry of Internal Affairs, cause of death database and included information on the date and cause of death.

### Covariates

Comorbidities were identified from medical notes recorded either three or more times in the outpatient department or one or more times in the inpatient department within 1 year before the index date for LT. The following comorbidities were identified among patients in our study cohort with ICD-9-CM codes: hypertension (ICD-9-CM codes: 401–405), hyperlipidemia (ICD-9-CM codes: 272.0–272.4), chronic kidney disease (CKD) (ICD-9-CM codes: 2504, 2741, 28311, 403, 404, 4401, 4421, 4473, 580, 581, 582, 583, 584, 585, 586, 587, 588, 589, 6421, and 6462), myocardial infarction (ICD-9-CM codes: 410 and 412), and congestive heart failure (ICD-9-CM codes: 398.91, 402.01, 402.11, 402.91, 404.01, 404.03, 404.11, 404.13, 404.91, 404.93, and 428). Immunosuppressant-use was defined as the use of calcineurin inhibitors, antimetabolic agents (purine antagonist), mammalian target of rapamycin (mTOR) inhibitors, and corticosteroids during hospitalization. The usage of antihypertensive agent for more than 90 days within 1 year before the date for LT was also recorded, including angiotensin-converting enzyme inhibitor (ACEI), angiotensin receptor blocker (ARB), calcium channel blocker (CCB), diuretics, β -blockers and α -blockers.

### Statistical Analysis

For baseline covariates, we used the analysis of variance and chi-square test to test continuous variables and category variables among three groups, respectively. To evaluate the risk of ESKD and death, we used the Cox proportional hazard models. In Cox proportional hazard models, we adjusted for potential confounders, such as age, sex, hypertension, hyperlipidemia, CKD, myocardial infarction, congestive heart failure, calcineurin inhibitors, antimetabolic agent (purine antagonist), mTOR inhibitors, corticosteroids, and antihypertensive agents to minimize confounding bias. We also assessed the assumption of proportional hazards for Cox proportional hazard models.

### Sensitivity Analyses

To deal with the immortal time bias, we did sensitivity analyses by using different study design (Supplementary Figures S3, S4). In the sensitivity analyses, all subjects were followed from the hospital discharge date for LT to the index date for ESKD, the occurrence of death, or until 31 December 2016 to evaluate the risk of ESKD. To estimate the risk of death, all subjects were followed from the hospital discharge date for LT to the occurrence of death or until 31 December 2016. We excluded patients who had developed ESKD before the index date for LT, but we did not exclude patients who had developed ESKD or died within 1 year after LT. Moreover, the time from the hospital discharge date for LT to the index date for PLTDM was accounted as the time in non-DM.

## Results

**FIGURE 1:**
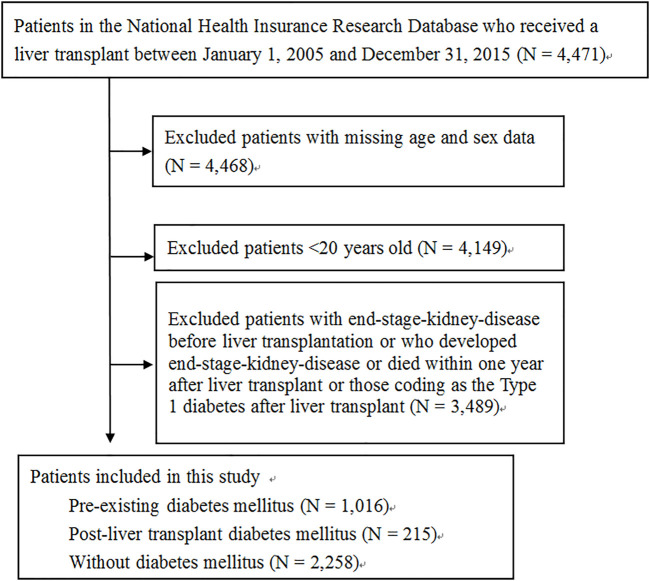
Flowchart of the selection criteria and process of selecting eligible patients.

A flowchart of the patient selection process is shown in Figure 1. After excluding patients with missing age and sex data, age <20 years old at the time of surgery, with ESKD before LT, who had been coding as the Type 1 diabetes after LT and who had developed ESKD or died within 1 year after LT, a total of 3,489 patients were included, of which, 1016 (29.1%) had pre-existing DM, 215 (6.2%) had PLTDM, and 2,258 (64.7%) did not have DM before or after LT. The distribution of demographic characteristics, comorbid medical disorders, and the use of immunosuppressant and antihypertensive agents for pre-existing DM group, PLTDM group, and without DM group are shown in Table 1. The mean age was higher in the pre-existing DM group (54.90 ± 7.28 years) than in the PLTDM (53.52 ± 7.99 years) and without DM groups (51.53 ± 9.34 years). Patients in the pre-existing DM group had a higher rate of comorbid medical disorders, including hypertension, hyperlipidemia, and CKD, than the PLTDM and without DM groups. For the use of immunosuppressants, including calcineurin inhibitor, antimetabolic agent (purine antagonist), mTOR inhibitors, corticosteroids and antihypertensive agents, including ACEI, ARB, CCB, diuretics, β -blockers and α -blockers, were reported.

**TABLE 1:** Characteristics of liver transplant patients.

Characteristic	DM	PLTDM	Non-DM	*p*-value
Number of patients	1,016	215	2,258	
Age, mean (SD), years	54.90 ± 7.28	53.52 ± 7.99	51.53 ± 9.34	<.0001
Age group, years, *N* (%)				<.0001
20–39	28 (2.8)	14 (6.5)	249 (11.0)	
40–59	699 (68.8)	150 (69.8)	1,568 (69.4)	
60–79	289 (28.4)	51 (23.7)	441 (19.5)	
Sex, male, *N* (%)	736 (72.4)	153 (71.2)	1,656 (73.3)	.7209
Comorbidities before the index date, *N* (%)				
Hypertension	362 (35.6)	38 (17.7)	345 (15.3)	<.0001
Hyperlipidemia	128 (12.6)	4 (1.9)	78 (3.5)	<.0001
Chronic kidney disease	179 (17.6)	20 (9.3)	190 (8.4)	<.0001
Myocardial infarction	5 (.5)	0 (.0)	2 (.1)	.0460
Congestive heart failure	15 (1.5)	3 (1.4)	19 (.8)	.2303
Treatment with drugs after liver transplant, *N* (%)				
Calcineurin inhibitors	1,004 (98.8)	214 (99.5)	2,229 (98.7)	.5729
Antimetabolic agent (Purine antagonist)	759 (74.7)	145 (67.4)	1,614 (71.5)	.0455
MTORIs	204 (20.1)	19 (8.8)	393 (17.4)	.0004
Corticosteroids	1,014 (99.8)	215 (100.0)	2,253 (99.8)	.7859
Treatment with drugs within 1 year prior to liver transplant, *N* (%)				
Antihypertensive agents	524 (51.6)	81 (37.7)	647 (28.7)	<.0001
ACEI	43 (4.2)	4 (1.9)	20 (0.1)	<.0001
ARB	132 (13.0)	8 (3.7)	83 (3.7)	<.0001
CCB	132 (13.0)	13 (6.1)	111 (4.9)	<.0001
Diuretic	582 (57.3)	135 (62.8)	1,033 (45.8)	<.0001
β-blockers	379 (37.3)	64 (29.8)	513 (22.7)	<.0001
α-blockers	15 (1.5)	2 (0.9)	10 (.4)	.0074
Hypoglycemic agent	731 (72.0)	—	—	—
Type I DM	74 (7.3)	0 (0.00)	—	—

Table 2 shows the incidence rate, crude and the adjusted hazard ratios (HRs) for ESKD among three groups during the 12-year follow-up. The incidence rates were 4.2, 8.1 and 13.1 per 1,000 person-years for without DM group, PLTDM group and pre-existing DM group, respectively. The crude HRs of ESKD for PLTDM group and pre-existing DM group were 1.92 (95% confidence interval [CI], .86–4.31, *p* = .1117) and 3.29 (95% confidence interval [CI], 2.12–5.10, *p* < .001) compared to without DM group, respectively. After adjustment for age, sex, hypertension, hyperlipidemia, CKD, myocardial infarction, congestive heart failure, calcineurin inhibitors, antimetabolic agent (purine antagonist), mTOR inhibitors, corticosteroids, and antihypertensive agents, the adjusted HRs of ESKD were 1.77 (95% CI, .78–3.99, *p* = .1694) and 2.61 (95% CI, 1.63–4.18, *p*< .001) for PLTDM group and pre-existing DM group compared to without DM group, respectively. We showed the models for ESKD and death in Supplementary Tables S1, S2, respectively.

**TABLE 2:** Incidence rate for end-stage renal disease.

	No. of event	Person-years	Incidence rate	Crude		Adjusted^a^	
				Hazard ratio (95% CI)	*p*-value	Hazard ratio (95% CI)	*p*-value
Non-DM	38	8,947	4.2	Ref.		Ref.	
PLTDM	7	860	8.1	1.92 (0.86–4.31)	.1117	1.77 (.78–3.99)	.1694
DM	43	3,279	13.1	3.29 (2.12–5.10)	<.0001	2.61 (1.63–4.18)	<.0001

a Adjustment: age, sex, hypertension, hyperlipidemia, chronic kidney disease, myocardial infarction, congestive heart failure, calcineurin inhibitors, antimetabolic agent (Purine antagonist), mTORIs and corticosteroids, antihypertensive agents.

For the risk of death, we show the incidence rate, the crude and the adjusted HRs among three groups during the 12-year follow-up in Table 3. The incidence rates were 31.6, 33.0 and 43.1 per 1,000 person-years for without DM group, PLTDM group and pre-existing DM group, respectively. The crude HRs were 1.05 (95% CI, .72–1.54, *p* = .7979) and 1.34 (95% CI, 1.10–1.64, *p* = .0045) for PLTDM group and pre-existing DM group compared to those without DM group, respectively. After adjustment for age, sex, hypertension, hyperlipidemia, CKD, myocardial infarction, congestive heart failure, calcineurin inhibitors, antimetabolic agent (purine antagonist), mTOR inhibitors, corticosteroids, and antihypertensive agents, the adjusted HRs were 1.05 (95% CI, .72–1.55, *p* = .7915) and 1.28 (95% CI, 1.04–1.59, *p* = .0204) for PLTDM group and pre-existing DM group compared to those without DM group, respectively.

**TABLE 3:** Incidence rate for death.

	No. of event	Person-years	Incidence rate	Crude		Adjusted^a^	
				Hazard ratio (95% CI)	*p*-value	Hazard ratio (95% CI)	*p*-value
Non-DM	285	9,032	31.6	Ref.		Ref.	
PLTDM	29	878	33.0	1.05 (.72–1.54)	.7979	1.05 (.72–1.55)	.7915
DM	145	3,362	43.1	1.34 (1.10–1.64)	.0045	1.28 (1.04–1.59)	.0204

a Adjustment: age, sex, hypertension, hyperlipidemia, chronic kidney disease, myocardial infarction, congestive heart failure, calcineurin inhibitors, antimetabolic agent (Purine antagonist), mTORIs and corticosteroids, antihypertensive agents.

Otherwise, we also performed the sensitivity analysis for the risk of ESKD and death, which is disclosed in Table 4. When compared to those without DM group, the adjusted HRs for the risk of ESKD and death were 1.70 (95% CI, .93–3.09, *p* = .0847) and 0.89 (95% CI, .69–1.14, *p* = .3383) for PLTDM group, and were 2.28 (95% CI, 1.51–3.43, *p* < .001) and 1.18 (95% CI, 1.01–1.39, *p* = .0373) for pre-existing DM group, respectively.

**TABLE 4:** Sensitivity analysis of risk of end-stage renal disease and death.

	No. of event	Person-years	Incidence rate	Crude		Adjusted^a^	
				Hazard ratio (95% CI)	*p*-value	Hazard ratio (95% CI)	*p*-value
End-stage renal disease							
Non-DM	47	10,021	4.7	Ref.		Ref.	
PLTDM	14	1,740	8.0	1.74 (0.96–3.16)	.0692	1.70 (.93–3.09)	.0847
DM	63	4,345	14.5	3.15 (2.15–4.60)	<.0001	2.28 (1.51–3.43)	<.0001
Death							
Non-DM	456	10,146	44.9	Ref.		Ref.	
PLTDM	73	1,770	41.2	.90 (.70–1.15)	.4104	.89 (.69–1.14)	.3383
DM	279	4,471	62.4	1.32 (1.14–1.53)	.0003	1.18 (1.01–1.39)	.0373

a Adjustment: age, sex, hypertension, hyperlipidemia, chronic kidney disease, myocardial infarction, congestive heart failure, calcineurin inhibitors, antimetabolic agent (Purine antagonist), mTORIs and corticosteroids, antihypertensive agents.

**FIGURE 2:**
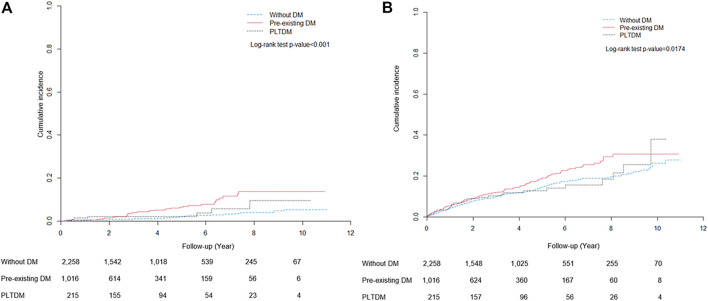
Cumulative incidence curves for **(A)** end-stage renal disease and **(B)** death.

The cumulative incidence curves for ESKD and death during the follow-up period are shown in Figure 2. The cumulative incidence of ESKD for pre-existing DM, PLTDM, and without DM groups were significantly different (Log-rank test *p* < .001). Patients with pre-existing DM had apparently higher incidence of ESKD than patients with PLTDM and without DM. For mortality, the cumulative incidence for pre-existing DM, PLTDM, and without DM groups were significantly different (Log-rank test *p* = .0174). Overall, pre-existing DM group had distinctly higher incidence of death than PLTDM and without DM group.

## Discussion

We performed a Nationwide population-based retrospective cohort study of patients who received an LT between 2005 and 2015 to evaluate the influence of DM on the risk of ESKD and mortality after LT. During the 12-year follow-up period, patients with pre-existing DM had a significantly higher risk of ESKD and mortality after LT. Patients with PLTDM did not increase the risk of ESKD and death after LT compared to those without DM. To our knowledge, this is the first study to examine the risk of ESKD and mortality after LT among patients with pre-existing DM, PLTDM and without DM.

DM is a group of metabolic diseases characterized by hyperglycemia, which is associated with microvascular and macrovascular complications resulting in long-term damage and failure of various organ systems (11). A case–control study that compared mortality rates after LT, including 57 patients with pre-existing DM (3, type I; 54, type II) and 114 age-, sex-, and race-matched patients without DM showed that 5-year survival was significantly lower in the DM group (34.4% vs. 67.7%, *p* = .002) (7). Here, the study population was large, and the results consistently showed that pre-existing DM reduces 12-year post-operation long-term survival for patients who have received LT.

In addition to pre-existing DM, PLTDM has emerged as a problem, which is diagnosed according to the 2003 International Consensus Guidelines. LT recipients who had no DM before transplantation but developed symptoms of DM with an elevated random plasma glucose (≥200 mg/dl) or an elevated fasting plasma glucose (≥126 mg/dl) or an elevated 2-h plasma glucose (≥200 mg/dl) during an oral glucose tolerance test were diagnosed to have PLTDM (12). PLTDM is a disorder with many risk factors, such as sex, use of immunosuppressants of the CNI family (tacrolimus and cyclosporine) or corticosteroid, pretransplant overweight, nonalcoholic steatohepatitis or hepatitis C infection, and type of liver donor (9). In this study, we found that 8.7% (215/2473) of patients who received LT developed PLTDM. The incidence of PLTDM was reported to range between 7.2% and 38% and may increase the risk of mortality and multiple morbidities (9). Studies have shown that PLTDM contributes to an increased risk of cardiovascular disease, infection, and transplant rejection, which negatively affects graft survival and survival (13, 14). In DM, elevated blood sugar levels are expected to increase cardiovascular risk and therefore mortality (15). Our results showed patients with PLTDM did not significantly reduce the long-term survival after LT during 12-years follow-up period when compared to those without DM. However, this relationship has not been clearly established due to the relatively few studies with small sample sizes. Two studies have reported conflicting results and shown that PLTDM is associated with an improved 5-year survival after LT, probably due to uncontrolled confounding factors (10, 16). Patients often present with cachexia and sarcopenia prior to LT (17). Studies have suggested that patients who recover from preoperative cachexia, sarcopenia, and malnutrition show better survival post LT (18). However, because the patients are recovering from malnutrition and gain weight, they may be at a greater risk of developing PLTDM (19), which may have better survival than those without DM only with good glycemic control after LT.

LT is associated with a deterioration of renal function in both early and late postoperative periods (20, 21). Kang et al. reported that renal function significantly decreased in the first year after LT (22). Kamei et al. showed that CKD developed in 26% of the patients with a median follow-up of 9.2 years after LT (23). Cohen et al. reported that 27.5% of the patients had severe renal dysfunction (measured GFR <40 ml/min/1.73 m^2^) at 5 years and the cumulative incidence of ESKD was 6.25% at 7 years and 10% at 10 years (6). Immunosuppressive treatment, essential for patients with LT to prevent graft rejection, is associated with nephrotoxicity, especially when calcineurin inhibitors are used. A study showed that a higher trough blood tacrolimus concentration correlated with reduced eGFR (24). However, we observed that no significant differences correlated with the use of immunosuppressive agents between the three groups. Previous studies have found that the etiologies of LT, such as hepatitis C infection and ethanol abuse, and donor type (circulatory death) may worsen renal function, increasing the prevalence of CKD after LT (24, 25). Our study shows that pre-existing DM is a significant risk factor for developing ESKD post-LT during a 12-year follow-up period, after adjustments for age, sex, co-morbidity, the usage of immunosuppressant, corticosteroid and antihypertensive agents.

As follow-up is started from date of transplant and PLTDM is defined at any time point during the first post-transplant year, this group will inevitably have survived until diagnosis of PLTDM and as such patients in this group will not be able to experience mortality until their diagnosis of PLTDM. This creates a biased low mortality rate in this group. In order to deal with the immortal time bias in the PLTDM group, we defined PLTDM as those who had been diagnosed of DM within 1-year after LT. We then excluded the patients who had developed ESKD or died within 1 year after LT and started follow-up 1 year after liver transplantation (all groups are defined at this time point as pre-existing DM, PLTDM, or non-DM). Furthermore, we also performed the sensitivity analysis for the risk of ESKD and death, which disclosed the consistent result that patients with pre-existing DM had a significantly higher risk of ESKD and mortality after LT during 12-year follow-up period.

Since the administrative health database have become more accessible, the validity of *ICD-9-CM* is crucial for the accuracy of the study. The *k* statistic, which assesses how well the administrative data set extracted from the electronic health record of *ICD-9-CM* agrees with actual chart review, confirmed substantial agreement in DM (*k* range from .7 to .8) and CKD patients (*k* > .8) (26, 27).

A major strength of this retrospective study is the relatively large number of patients with long follow-up periods. We have established the risk of developing ESKD and long-term survival in patients with pre-existing DM and PLTDM and without DM after LT. However, this study has some limitations. First, our results were based on a retrospective cohort study. The NHIRD is a secondary database and information on medical examination data, laboratory data, detailed rejection condition, transient hyperglycemia condition post-LT and the etiology of DM, CKD, death, and LT was not provided by the administrative database. PLTDM can be defined as a degree of hyperglycemia after LT. A reliable diagnosis of PLTDM must be made after the doses of immunosuppressive agents or steroid have been tapered and are stable. In our study, we defined PLTDM as those patients who had been diagnosed of DM within 1-year post-LT and it may create bias of inevitably included post-LT transient hyperglycemia. Second, our cohort study included patients from a 12-year period, and variations in the type of liver donor and the selection criteria for LT may influence long-term outcome. Thirds, ESKD takes times to develop, therefore, if PLTDM affect the outcome of ESKD, it may need longer follow-up period to elucidate the difference. We recommend extending the follow-up period and conducting further prospective studies to clarify long-term outcomes due to the conflicting survival rates reported between patients with PLTDM and without DM. Last, the power of the model for ESKD may not be large enough when we included many covariates in the model. We recommend to included more patients in the LT cohort in the future study.

In conclusion, this study demonstrated that patients with pre-existing DM had a significantly higher risk of developing ESKD and increasing the risk of death**.** PLTDM did not increase the risks of ESKD and death compared to those without DM. We emphasize the need for adequately powered studies and extending the follow-up period to explore the long-term outcome of PLTDM.

## Capsule Summary Sentence

Improvements in early post-liver transplantation (LT) survival rates have increased the importance of understanding the risks factors for late post-LT morbidity and mortality. This retrospective study aimed to investigate the effect of diabetes mellitus (DM) on the risks of end-stage kidney disease (ESKD) and post-LT mortality. This study demonstrated that patients with pre-existing DM had a significantly higher risk of developing ESKD and increasing the risk of death compared to without DM group. We emphasize the need for adequately powered studies to explore the role of Post-LT DM (PLTDM) to explain post-LT morbidity and mortality.

## Data Availability

The data underlying this article were provided by National Health Insurance Research Database in Taiwan under license/by permission. The datasets presented in this article are not readily available because it is a restricted database only accessible by formal application to the Health and Welfare Data Science Center of Taiwan. Requests to access the datasets should be directed to the Health and Welfare Data Science Center of Taiwan (http://dep.mohw.gov.tw/DOS/cp-5119-59201-113.html).

## Supplementary Material

Supplementary Figure 1 Main study design for ESKD.

Supplementary Figure 2 Main study design for death.

Supplementary Figure 3 Study design in sensitivity analysis for ESKD.

Supplementary Figure 4 Study design in sensitivity analysis for death.

## Abbreviations

ACEI, angiotensin-converting enzyme inhibitor; ARB, angiotensin receptor blocker; CCB, calcium channel blocker; CI, confidence interval; CKD, chronic kidney disease; DM, diabetes mellitus; ESKD, end-stage kidney disease; HRs, hazard ratios; ICD-9-CM, International Classification of Disease, Revision 9, Clinical Modification; LT, liver transplantation; mTOR, mammalian target of rapamycin; NHI, National Health Insurance; NHIRD, National Health Insurance Research Database; PLTDM, post-liver transplantation diabetes mellitus; Post-LT, post-liver transplantation.

## References

[1] Allen AM Kim WR Therneau TM Larson JJ Heimbach JK Rule AD Chronic Kidney Disease and Associated Mortality after Liver Transplantation - A Time-dependent Analysis Using Measured Glomerular Filtration Rate. J Hepatol (2014) 61(2):286–92. 10.1016/j.jhep.2014.03.034 24713190 PMC4160310

[2] Stepanova M Wai H Saab S Mishra A Venkatesan C Younossi ZM The Outcomes of Adult Liver Transplants in the United States from 1987 to 2013. Liver Int (2015) 35(8):2036–41. 10.1111/liv.12779 25559873

[3] Huang Y-Y Hsu C-C Chou C-L Loong C-C Wu M-S Chou Y-C Trends in the Use of Maintenance Immunosuppressive Drugs Among Liver Transplant Recipients in Taiwan: a Nationwide Population-Based Study. Pharmacoepidemiol Drug Saf (2016) 25(6):661–7. 10.1002/pds.3964 26799240

[4] Ojo AO Held PJ Port FK Wolfe RA Leichtman AB Young EW Chronic Renal Failure after Transplantation of a Nonrenal Organ. N Engl J Med (2003) 349(10):931–40. 10.1056/nejmoa021744 12954741

[5] Pawarode A Fine DM Thuluvath PJ. Independent Risk Factors and Natural History of Renal Dysfunction in Liver Transplant Recipients. Liver Transplant (2003) 9(7):741–7. 10.1053/jlts.2003.50113 12827563

[6] Cohen AJ Stegall MD Rosen CB Wiesner RH Leung N Kremers WK Chronic Renal Dysfunction Late after Liver Transplantation. Liver Transplant (2002) 8(10):916–21. 10.1053/jlts.2002.35668 12360433

[7] John P Thuluvath PJ. Outcome of Liver Transplantation in Patients with Diabetes Mellitus: a Case-Control Study. Hepatology (2001) 34(5):889–95. 10.1053/jhep.2001.29134 11679959

[8] Wallia A Parikh ND Molitch ME Mahler E Tian L Huang JJ Posttransplant Hyperglycemia Is Associated with Increased Risk of Liver Allograft Rejection. Transplantation (2010) 89(2):222–6. 10.1097/tp.0b013e3181c3c2ff 20098286 PMC2946243

[9] Peláez-Jaramillo MJ Cárdenas-Mojica AA Gaete PV Mendivil CO Post-liver Transplantation Diabetes Mellitus: a Review of Relevance and Approach to Treatment. Diabetes Ther (2018) 9(2):521–43. 10.1007/s13300-018-0374-8 29411291 PMC6104273

[10] Darstein F König C Hoppe-Lotichius M Grimm D Knapstein J Zimmermann A New Onset of Diabetes after Transplantation Is Associated with Improved Patient Survival after Liver Transplantation Due to Confounding Factor. Eur J Intern Med (2015) 26(6):439–44. 10.1016/j.ejim.2015.05.018 26058989

[11] Chawla R Chawla A Jaggi S Microvasular and Macrovascular Complications in Diabetes Mellitus: Distinct or Continuum? Indian J Endocr Metab (2016) 20(4):546. 10.4103/2230-8210.183480 PMC4911847 27366724

[12] Davidson J Wilkinson A Dantal J Dotta F Haller H Hernández D New-onset Diabetes after Transplantation: 2003 International Consensus Guidelines. Proceedings of an International Expert Panel Meeting. Barcelona, Spain, 19 February 2003Proceedings of an International Expert Panel Meeting. Barcelona, Spain, 19 February 2003. Transplantation (2003) 75(10 Suppl. l):SS3–24. 10.1097/01.TP.0000069952.49242.3E 12775942

[13] Moon JI Barbeito R Faradji RN Gaynor JJ Tzakis AG Negative Impact of New-Onset Diabetes Mellitus on Patient and Graft Survival after Liver Transplantation: Long-Term Follow up. Transplantation (2006) 82(12):1625–8. 10.1097/01.tp.0000250361.60415.96 17198248

[14] Anderson A Lewis D Steinke D Ranjan D Smith K Clifford T Effects of Hyperglycemia on the Development of New-Onset Diabetes after Liver Transplantation. Prog Transplant (2009) 19(4):298–303. 10.7182/prtr.19.4.wq67603t74587q65 20050451

[15] Chowdhury EK Owen A Ademi Z Krum H Johnston CI Wing LMH Short- and Long-Term Survival in Treated Elderly Hypertensive Patients with or without Diabetes: Findings from the Second Australian National Blood Pressure Study. Am J Hypertens (2014) 27(2):199–206. 10.1093/ajh/hpt212 24249722

[16] Liu FC Lin JR Chen HP Tsai YF Yu HP. Prevalence, Predictive Factors, and Survival Outcome of New-Onset Diabetes after Liver Transplantation: a Population-Based Cohort Study. Medicine (Baltimore) (2016) 95(25):e3829. 10.1097/MD.0000000000003829 27336869 PMC4998307

[17] Srinivasan D. Consilience in Sarcopenia of Cirrhosis. J Cachexia, Sarcopenia Muscle (2012) 3(4):225–37. 10.1007/s13539-012-0069-3 22648736 PMC3505573

[18] Lim S Kim KM Kim MJ Woo SJ Choi SH Park KS The Association of Maximum Body Weight on the Development of Type 2 Diabetes and Microvascular Complications: MAXWEL Study. PLoS One (2013) 8(12):e80525. 10.1371/journal.pone.0080525 24324607 PMC3851456

[19] DiMartini A Cruz RJ Jr Dew MA Myaskovsky L Goodpaster B Fox K Muscle Mass Predicts Outcomes Following Liver Transplantation. Liver Transpl (2013) 19(11):1172–80. 10.1002/lt.23724 23960026 PMC4382961

[20] Li Y Li B Wang W Lv J. Risk Factors for New-Onset Chronic Kidney Disease in Patients Who Have Received a Liver Transplant. Exp Ther Med (2018) 15(4):3589–95. 10.3892/etm.2018.5823 29545888 PMC5840952

[21] Weber ML Ibrahim HN Lake JR Renal Dysfunction in Liver Transplant Recipients: Evaluation of the Critical Issues. Liver Transpl (2012) 18(11):1290–301. 10.1002/lt.23522 22847917

[22] Kang G Lee I Ahn K Kim J Kwak S Choi D, editors. One-Year Follow-Up of the Changes in Renal Function after Liver Transplantation in Patients without Chronic Kidney Disease. Transplantation Proceedings. Elsevier (2016). 10.1016/j.transproceed.2016.02.013 27320584

[23] Kamei H Onishi Y Nakamura T Ishigami M Hamajima N Role of Cytokine Gene Polymorphisms in Acute and Chronic Kidney Disease Following Liver Transplantation. Hepatol Int (2016) 10(4):665–72. 10.1007/s12072-016-9721-x 27003899

[24] Gojowy D Kubis P Gorecka M Karkoszka H Wiecek A Adamczak M. Chronic Kidney Disease in Patients after Liver Transplantation: A Long-Term Retrospective Analysis from 1 Transplantation Center. Transpl Proc (2020) 52(8):2492–6. 10.1016/j.transproceed.2020.02.081 32249052

[25] Cacoub P Desbois AC Isnard-Bagnis C Rocatello D Ferri C Hepatitis C Virus Infection and Chronic Kidney Disease: Time for Reappraisal. J Hepatol (2016) 65(1):S82–S94. 10.1016/j.jhep.2016.06.011 27641990

[26] Khokhar B Jette N Metcalfe A Cunningham CT Quan H Kaplan GG Systematic Review of Validated Case Definitions for Diabetes in ICD-9-Coded and ICD-10-Coded Data in Adult Populations. BMJ open (2016) 6(8):e009952. 10.1136/bmjopen-2015-009952 PMC4985868 27496226

[27] Navaneethan SD Jolly SE Schold JD Arrigain S Saupe W Sharp J Development and Validation of an Electronic Health Record-Based Chronic Kidney Disease Registry. Cjasn (2011) 6(1):40–9. 10.2215/cjn.04230510 21051745 PMC3022247

