# Supplementary figures and images for: The Influence of Diabetes Mellitus on the Risks of End-Stage Kidney Disease and Mortality After Liver Transplantation
Source: Transpl Int. 2022 Feb 7;35:10023. doi: 10.3389/ti.2022.10023 (PMC8842258; doi:10.3389/ti.2022.10023)

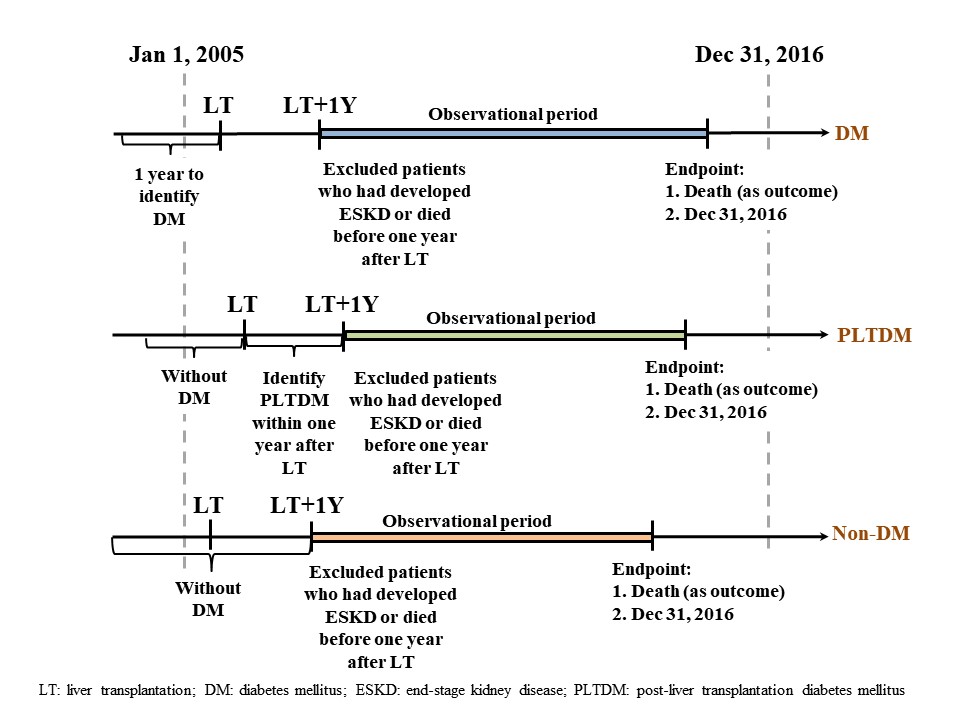

Supplement: Supplementary file 1 [file Image3.JPEG]

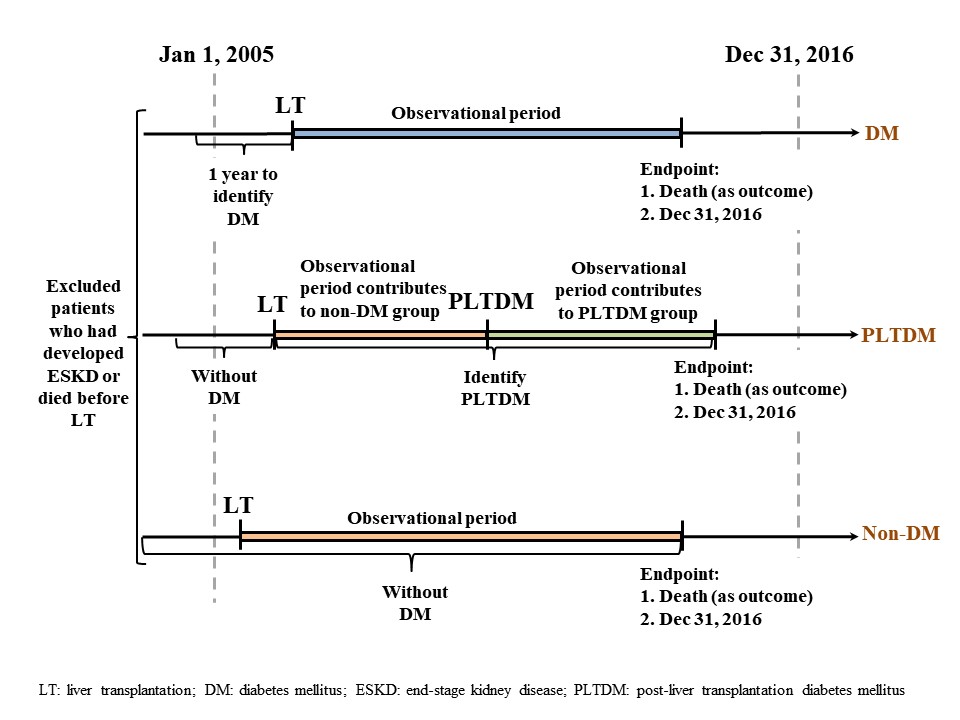

Supplement: Supplementary file 2 [file Image1.JPEG]

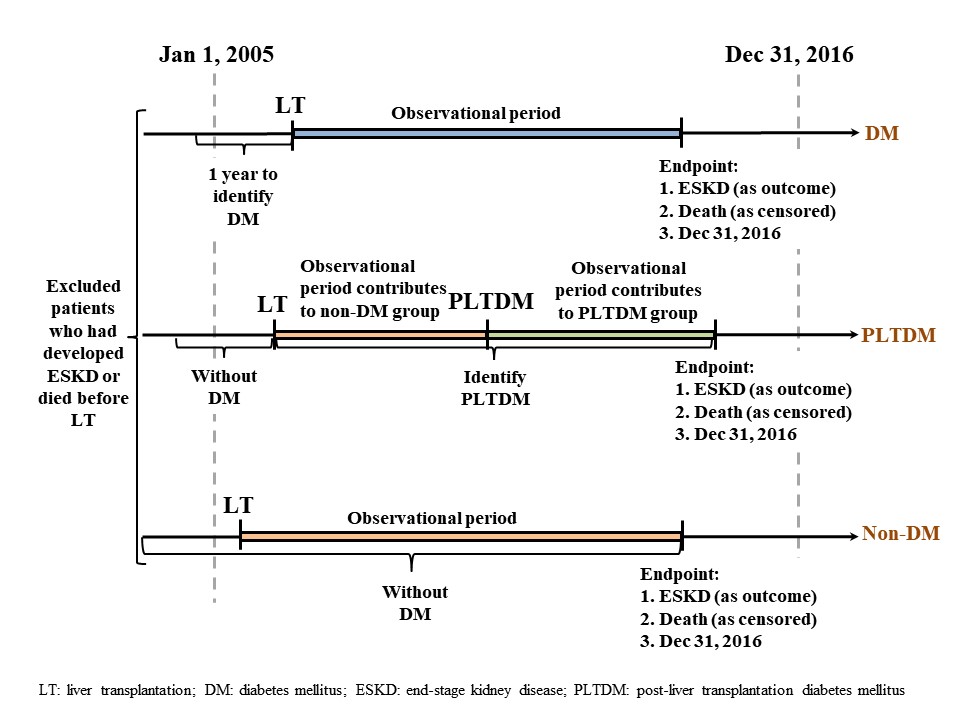

Supplement: Supplementary file 3 [file Image4.JPEG]

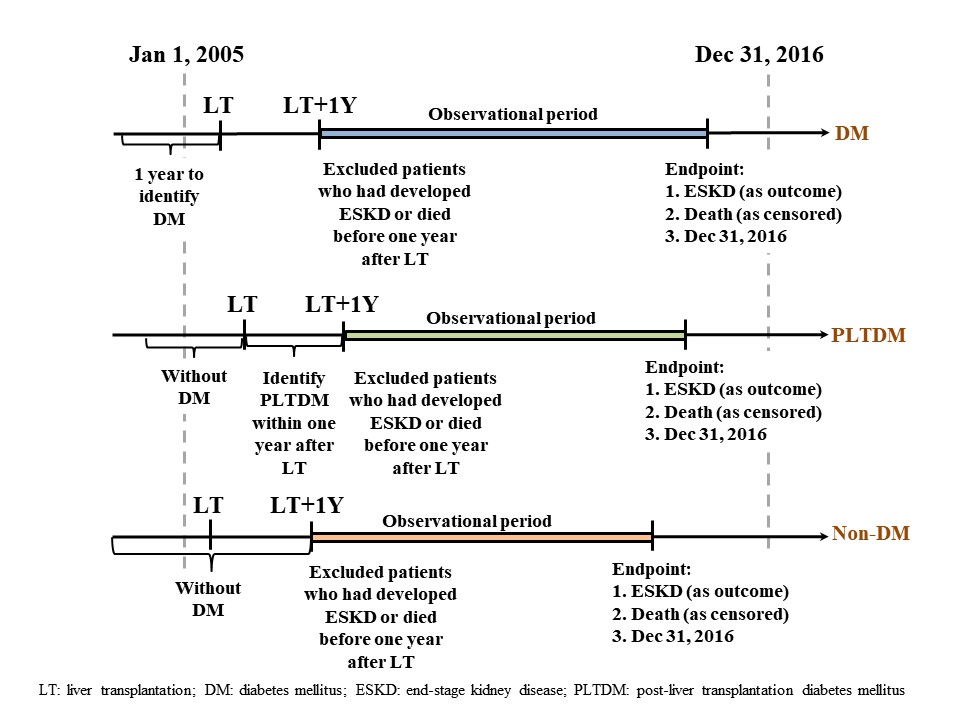

Supplement: Supplementary file 4 [file Image2.JPEG]
