# Supplementary material for: The Influence of Diabetes Mellitus on the Risks of End-Stage Kidney Disease and Mortality After Liver Transplantation
Source: Transpl Int. 2022 Feb 7;35:10023. doi: 10.3389/ti.2022.10023 (PMC8842258; doi:10.3389/ti.2022.10023)
Supplement: Supplementary file 5 [file DataSheet1.docx]

**Supplementary Tables**

**Supplementary Table 1 The model for end-stage renal disease**

| Characteristic | Hazard Ratio  (95% CI) | P-value |
| --- | --- | --- |
| PTDM vs non-DM | 1.77 (0.78-3.99) | 0.169 |
| DM vs non-DM | 2.61 (1.63-4.18) | <.001 |
| Age, years | 1.01 (0.99-1.04) | 0.325 |
| Sex, Male | 1.07 (0.65-1.75) | 0.795 |
| Calcineurin inhibitors | 1.23 (0.30-5.07) | 0.773 |
| Antimetabolic agent (Purine antagonist) | 0.55 (0.36-0.85) | 0.007 |
| mTORIs | 1.85 (1.06-3.23) | 0.031 |
| Corticosteroids | 0.49 (0.06-3.84) | 0.499 |
| Hypertension | 1.57 (0.93-2.67) | 0.093 |
| Hyperlipidemia | 0.49 (0.15-1.57) | 0.230 |
| Chronic Kidney Disease | 4.43 (2.83-6.95) | <.001 |
| Myocardial infarction | <.001 (<.001) | 0.988 |
| Congestive heart failure | <.001 (<.001) | 0.983 |
| Antihypertensive agents | 0.85 (0.52-1.37) | 0.498 |

**Supplementary Table 2 The model for death**

| Characteristic | Hazard Ratio  (95% CI) | P-value |
| --- | --- | --- |
| PTDM vs non-DM | 1.28 (1.04-1.59) | 0.020 |
| DM vs non-DM | 1.05 (0.72-1.55) | 0.792 |
| Age, years | 1.02 (1.01-1.03) | 0.001 |
| Sex, Male | 1.06 (0.86-1.30) | 0.613 |
| Calcineurin inhibitors | 0.86 (0.44-1.69) | 0.669 |
| Antimetabolic agent (Purine antagonist) | 0.82 (0.68-1.00) | 0.052 |
| mTORIs | 1.63 (1.28-2.06) | <.001 |
| Corticosteroids | 1.15 (0.16-8.46) | 0.894 |
| Hypertension | 1.01 (0.78-1.30) | 0.966 |
| Hyperlipidemia | 1.02 (0.68-1.54) | 0.926 |
| Chronic Kidney Disease | 1.05 (0.78-1.41) | 0.768 |
| Myocardial infarction | 1.83 (0.45-7.49) | 0.399 |
| Congestive heart failure | 0.81 (0.30-2.20) | 0.683 |
| Antihypertensive agents | 0.85 (0.68-1.05) | 0.138 |
